# The impact of within-host coinfection interactions on between-host parasite transmission dynamics varies with spatial scale

**DOI:** 10.1098/rspb.2024.0103

**Published:** 2024-04-17

**Authors:** Shaun P. Keegan, Amy B. Pedersen, Andy Fenton

**Affiliations:** ^1^ Department of Evolution, Ecology and Behaviour, Institute of Infection, Veterinary and Ecological Sciences, University of Liverpool, Liverpool L69 7ZB, UK; ^2^ Institute of Ecology and Evolution, School of Biological Sciences, University of Edinburgh, Edinburgh EH9 3FL, UK

**Keywords:** disease ecology, parasite ecology, spatial ecology, disease spread, coinfection, parasite interactions

## Abstract

Within-host interactions among coinfecting parasites can have major consequences for individual infection risk and disease severity. However, the impact of these within-host interactions on between-host parasite transmission, and the spatial scales over which they occur, remain unknown. We developed and apply a novel spatially explicit analysis to parasite infection data from a wild wood mouse (*Apodemus sylvaticus*) population. We previously demonstrated a strong within-host negative interaction between two wood mouse gastrointestinal parasites, the nematode *Heligmosomoides polygyrus* and the coccidian *Eimeria hungaryensis*, using drug-treatment experiments. Here, we show this negative within-host interaction can significantly alter the between-host transmission dynamics of *E. hungaryensis*, but only within spatially restricted neighbourhoods around each host. However, for the closely related species *E. apionodes*, which experiments show does not interact strongly with *H. polygyrus*, we did not find any effect on transmission over any spatial scale. Our results demonstrate that the effects of within-host coinfection interactions can ripple out beyond each host to alter the transmission dynamics of the parasites, but only over local scales that likely reflect the spatial dimension of transmission. Hence there may be knock-on consequences of drug treatments impacting the transmission of non-target parasites, altering infection risks even for non-treated individuals in the wider neighbourhood.

## 1. Introduction

Most individuals, among wildlife, livestock and humans in many parts of the world, are infected by multiple parasite species throughout their lives [1]. It is well known that such coinfecting parasites can interact strongly with each other within individual coinfected hosts [2–7], with potentially important implications for host susceptibility, clinical disease progression, and treatment and vaccine efficacy [8]. However, most attention to date has focused primarily on the mechanisms driving within-host interactions, and the effects on the individual hosts they occur within, with little understanding of the consequences these interactions have for parasite transmission between hosts, or for the role they play in driving spatio-temporal and epidemiological dynamics of infection across larger spatial scales.

Intuitively, we may expect within-host coinfection interactions to impact parasite population dynamics through a process of ‘transmission modification’, whereby coinfection alters the production of parasite transmission stages, thereby modifying the risk of other hosts in the population becoming infected. Existing theory broadly supports this hypothesis, but also suggests that the nature of the scaling relationship from within-host interaction to population-level impact may be highly nonlinear, dependent, at least in part, on the mechanism underlying the within-host interaction [9–11]. Some evidence from natural systems supports these predictions. For example, contrasting individual- and population-level effects of helminth–bovine tuberculosis (bTB) coinfection have been predicted in African buffalo [12], whereby anthelmintic treatment benefited individuals through reduced bTB-induced host mortality, but this was predicted to increase bTB transmission at the population level [12]. Furthermore, analyses of specific gastrointestinal nematode and protozoan infections in UK wood mice (*Apodemus sylvaticus*) find no signal of interspecific associations at the host population scale [13], even though drug treatment experiments in wild populations and controlled coinfection experiments in the laboratory clearly show the nematodes strongly suppress the protozoans within individual coinfected hosts [4,14,15]. Together, these results indicate that within-host coinfection interactions can have potentially counterintuitive effects on parasite transmission through the host population.

We suggest that a key aspect missing from coinfection studies to date, that prevents clear assessment of the impact of within-host interactions on between-host transmission dynamics, is spatial scale. Most studies either look exclusively at the within-host scale (e.g. through controlled infection/coinfection experiments on individual hosts), or the whole-population scale (e.g. looking for patterns of parasite co-association across the entire host population). Yet parasite transmission is an inherently spatial process: the movement of a parasite from one individual to another typically requires a degree of spatial proximity, the magnitude of which may vary with transmission mode. Hence, we may expect the effects of transmission modification due to coinfection interactions to show strong spatial structuring. To date, however, there has been no explicit empirical assessment of how within-host parasite interactions affect parasite transmission in a natural system, nor quantification of the spatial scale over which such effects occur.

We present a spatially explicit analysis of the between-host transmission consequences of within-host interactions among three coinfecting parasites in a wild wood mouse population. Through anthelmintic drug treatment experiments in these natural populations, we have previously shown that the dominant gastrointestinal (GI) nematode, *Heligmosomoides polygyrus*, suppresses coinfections by the coccidial parasite *Eimeria hungaryensis* [4], both highly prevalent GI parasites of wood mice that transmit via eggs/oocysts released from infected animals, which form pools of relatively long-lived infective stages in the environment. Our previous individual-level experiments have shown that animals treated with the anti-nematode drug Ivermectin showed a 15-fold increase in *E. hungaryensis* oocyst shedding one to three weeks post-treatment, compared with untreated animals. Hence, *H. polygyrus* interacts negatively with *E. hungaryensis*, suppressing output of its infective stages from coinfected animals. Notably, however, there was little impact of treatment on the closely related species, *E. apionodes* [4], suggesting that *H. polygyrus* has little impact on this species in coinfected mice. The hypothesized reason for this difference between the two *Eimeria* species arises from the fact the species differ in their preferential infection niches in the gut: *E. hungaryensis* and *H. polygyrus* both infect in the same part of the small intestine, specifically the duodenum, whereas *E. apionodes* infects further down the small intestine, in the lower ileum [4,15]. Hence, the impact of *H. polygyrus* on coinfecting *Eimeria* is highly localized within the gut, due either to competition for shared resources or physical interference (e.g. disruption of epithelial tissue integrity and/or reduction in the availability or longevity of gut epithelial cells), or localized immune responses (e.g. reduction in specific and total antibodies during coinfection [4,16]), that have little impact on *E. apionodes* further down the gut.

The contrasting *H. polygyrus*-mediated within-host coinfection effects between these two closely related *Eimeria* species with otherwise very similar life cycles provides an ideal opportunity to compare the population-dynamic consequences of the within-host interaction. Specifically, we hypothesize that increasing neighbourhood-wide prevalence and/or mean abundance of *H. polygyrus*-infected animals will, through coinfection-mediated transmission modification, reduce the local force of infection of *E. hungaryensis* sufficiently to reduce *E. hungaryensis* infection intensity in focal animals within the neighbourhood; however, we predict to find no such effect for *E. apionodes*. Given the experiment reported in Knowles *et al.* [4] did not detect an effect of treatment on *Eimeria* presence/absence however [4], we hypothesize that we are unlikely to see a neighbourhood-wide effect of *H. polygyrus* on *Eimeria* infection probability (presence/absence) in focal animals.

We also hypothesize that the magnitude of neighbourhood-wide effects of *H. polygyrus* on focal *Eimeria* infections will vary in both time and space, reflecting the spatio-temporal scales of transmission of these faecal-oral parasites. In particular, we hypothesize that transmission modification will be most apparent at local spatial scales and over time scales reflecting the deposition, development and uptake of infective stages from the environment. We test these hypotheses through a spatially explicit analyses of wood mouse trapping data over a range of neighbourhood sizes, and show that within-host coinfection interactions do indeed influence between-host transmission of *E. hungaryensis*, but not *E. apionodes*, but the effects for *E. hungaryensis* are strongest over relatively localized spatial scales around individual hosts.

## 2. Material and methods

### (a) Wood mouse and parasite data collection

Individual wood mice (*A. sylvaticus*) were trapped every two weeks, using baited Sherman traps (Alana Ecology, UK; dimensions 8.9 cm × 7.6 cm × 22.9 cm), from May to December 2012 in Haddon Wood, England (Wirral Peninsula, 51.0508° N, 3.4980° W). Traps were laid on a semi-permanent 110 × 110 m grid, with two traps every 10 m, which were checked every day for 3 consecutive days, every two weeks. At first capture, all mice were permanently tagged with a subcutaneous microchip transponder for identification (AVID Friend Chip). For each mouse at every capture, we took the following metrics: body length (nose tip to base of tail in mm), weight (g), sex, reproductive status and an estimate of age (see [4] for additional methodological details). At every capture, we also collected faecal samples from previously sterilized, single occupancy traps for faecal floatation and microscopic analysis to identify and quantify both helminth eggs and coccidial oocysts (measured as eggs or oocysts/gram of faeces examined). As part of an on-going experiment, a subset of animals received either a weight-adjusted dose of Ivermectin (an anthelmintic known to reduce nematodes: Eqvalan, 9.4 mg kg^−1^; *n* = 68 animals treated) or Vecoxan (an anti-coccidial treatment; *n* = 63 animals treated) or both (*n* = 57 animals) each time they were caught, from their first capture onwards; all other animals (*n* = 70) received water as a control. There were 986 captures in total. All mice were released at the point of capture after handling. See electronic supplementary material and [4] for more details for field and laboratory methods.

### (b) Neighbourhood analysis: data structure

**Figure 1.**
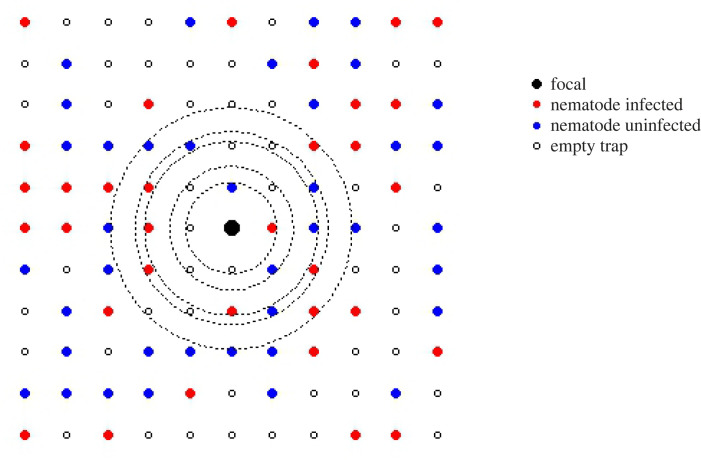
Example trapping grid with wood mouse neighbourhoods. This trapping grid shows the relative locations of traps, with the current focal animal shown in black. Neighbouring traps are colour-coded either red for an animal infected with the nematode *H. polygyrus*, blue for an uninfected animal, or blank for no capture within the specified time window of the focal's capture. Five example neighbourhoods of increasing size around the focal are shown in dotted outline. The prevalence of nematode infection within each neighbourhood size of the focal is then calculated as the proportion of infected animals (red points) out of the total number of animals caught (red + blue points) within that neighbourhood, for the specified time window.

We considered each individual at a single capture point as the ‘focal’ individual in turn. Around each focal animal at each capture, we defined its neighbourhood of size *r* as the Euclidean distance of all trap locations lying within *r* metres of the focal's capture location, from *r* = 10 m (comprising the four closest neighbours only around each focal capture) up to *r* = 50 m (approximately half the trapping grid) (figure 1). Note, this results in a list of 13 unique neighbourhood sizes, accounting for all possible distances from a single capture point to other points within a 50 m radius (10 m, 14.1 m, 20 m, 22.4 m, 28.3 m, 30 m, 31.6 m, 36.1 m, 40 m, 41.2 m, 42.4 m, 44.7 m, 50 m). Initial analyses suggested neighbourhood size effects largely stabilize after 40 m, so we removed the entries between 40 and 50 m to limit computational runtime at those larger distances. We then determined the number of other individuals (neighbours) caught at traps within that neighbourhood within a specified time window prior to the capture date of the focal individual; animals caught after the focal's capture date were ignored, as they could not have contributed to the focal individual's infection status at that time point, and animals caught prior to the specified time window were deemed unlikely to have contributed current infection levels in the focal host. We explored sensitivity of our findings to the duration of this window by running our analyses to a range of time window sizes (up to 17 days, 34 days, 51 days, or all previous time points prior to the focal's capture date; date ranges were dictated by trapping frequency, but broadly reflect two weeks, one month, two months or no limit to prior space use by neighbours).

For each specified time window and neighbourhood size around each focal individual, we calculated both the proportion of neighbouring hosts that were infected with the nematode *H. polygyrus* (neighbourhood prevalence) and the mean abundance of *H. polygyrus* infection (neighbourhood abundance) within each neighbourhood. For some individuals and neighbourhood sizes, some of their potential neighbourhood lay outside the experimental grid. In these instances, we assumed that the grid was representative of the wider woodland, and as such the observed prevalence and mean abundance of *H. polygyrus* infection within the neighbourhood on the grid reflected that of the focal's entire neighbourhood. We also scaled the number of neighbours caught around each focal by the inverse of the proportion of the focal's neighbourhood lying on the grid, to estimate the total number of neighbours around each focal.

### (c) Neighbourhood analysis: statistical analyses

We analysed the above datasets for each neighbourhood size (*r* = 10 m, 14.1 m, 20 m, 22.4 m, 28.3 m, 30 m, 31.6 m, 36.1 m, 40 m, 50 m, as described above) and time window (up to 17 days, 34 days, 51 days, or all previous time points prior to the focal's capture date), to determine whether there was a signal of increasing neighbourhood-level *H. polygyrus* infections on each focal individual's infection status with either *E. hungaryensis* or *E. apionodes*. We repeated these analyses for all four combinations of measurements of neighbourhood-wide *H. polygyrus* infection status (prevalence or mean abundance of infection) and focal *Eimeria* infection status (intensity or presence/absence). A significant effect of neighbourhood *H. polygyrus* prevalence or abundance on focal *Eimeria* infection status (presence/absence or intensity) would suggest that the within-host effects of *H. polygyrus* coinfection among animals in the wider neighbourhood modify oocyst shedding of that *Eimeria* species sufficiently to alter within-neighbourhood transmission, thereby impacting on *Eimeria* infection levels of focal animals within that neighbourhood.

For each time window and neighbourhood size, we ran generalized linear mixed effects models (GLMMs) implemented in a Bayesian framework using stan (R packages: rstan (version 2.17.3), brms (version 2.3.0), five chains per model, 4000 iterations per chain, uninformative normal priors (mean = 0, s.d. = 10)). Each individual's *Eimeria* infection status (either *E. hungaryensis* or *E. apionodes* in turn) was quantified either as log infection intensity (number of oocysts per gram of faeces among infected animals) as the response variable in a Gaussian GLMM, or as presence/absence in a Bernoulli GLMM. Our main fixed effect of interest was a measure of the neighbourhood-level infection status of *H. polygyrus*, quantified either as neighbourhood prevalence (proportion of hosts infected), or neighbourhood mean abundance (mean eggs per gram of all hosts), within the specified neighbourhood size around the focal individual, and within the specified time window.

In all models, we controlled for the following potential confounding variables as fixed effects: the focal animal's *H. polygyrus* infection status (positive/negative), sex (male or female) and age (adult or not); the total number of animals in the defined neighbourhood, the neighbourhood-wide prevalence of the relevant *Eimeria* species, and the date of capture as a second-order polynomial to account for nonlinear seasonal effects. For simplicity, we did not include any interactions between these terms. We also included the focal animal's ID (unique PIT tag number) as a random effect to control for potential pseudo-replication arising from multiple captures of the same individual. Hence, our statistical models took the general form:

$$\begin{array}{rl} & \mathrm{focal}\;Eimeria\;\mathrm{status}\;∼\;\mathrm{neighbourhood}\;H.\;polygyrus\;\mathrm{status}+\mathrm{focal}\;H.\;polygyrus\;\mathrm{status}+\mathrm{focal}\;\mathrm{sex}\\ & \;+\mathrm{focal}\;\mathrm{age}+\mathrm{neighbourhood}\;Eimeria\;\mathrm{prevalence}+\mathrm{neighbourhood}\;\mathrm{mouse}\;\mathrm{abundance}\\ & \;+\mathrm{poly}(\mathrm{capture}\;\mathrm{date},\;2)+(1|\mathrm{ID}) \end{array}$$

We did not carry out any model reduction or simplification, to ensure consistency in model structure across all analyses, and we report the effect sizes (median GLMM coefficients, ± 95% credible intervals) for neighbourhood-wide *H. polygyrus* status (prevalence or mean abundance) on focal host *E. hungaryensis* or *E. apionodes* status (infection intensity or presence/absence), for each neighbourhood size and time window, while controlling for the same set of potential confounders in all analyses. Negative effect sizes would suggest that increasing prevalence or mean abundance of *H. polygyrus* in the neighbourhood was associated with a reduction in the presence or intensity of *Eimeria* infection of focal individuals within the specified time window and neighbourhood size.

We note that our focus is on how *H. polygyrus* infections within a specified neighbourhood size relate to *Eimeria* infections in each focal host. As such, increasing neighbourhood sizes around each focal individual incorporated the same hosts that were found within smaller neighbourhoods around that focal individual. Hence successive neighbourhoods are not independent of each other. Due to that non-independence, we did not seek to include all possible neighbourhoods in a single analysis, with a model term describing how the effect of *H. polygyrus* changes with neighbourhood size. Rather, we conducted a series of analyses to understand how changing spatial scale altered the association between neighbourhood *H. polygyrus* and focal *Eimeria* infections. This approach requires multiple testing through repeated GLMM analyses. For this reason, in part, we did not evaluate statistical significance of effects through the calculation of *p*-values for each test. Instead, we adopted the above Bayesian approach to generate a series of estimated effect sizes, which we use to infer the consequence of increasing *H. polygyrus* infections in the neighbourhood on focal *Eimeria* infection levels at each neighbourhood size.

To ensure that the above method is not biased to generating associations when there are none, we carried out a randomization test, whereby we reran the analyses on a dataset that randomly assigned observed pairs of *H. polygyrus* and *E. hungaryensis* infection data from each animal to the spatial and temporal capture records of other animals in the dataset. That is, we retained individual-level *H. polygyrus* and *E. hungaryensis* associations, but detached those from their neighbourhood context. Hence this approach tests, for realistic host spatio-temporal captures, and parasite (co)infection data, whether our neighbourhood analysis method generates spurious associations between *H, polygyrus* neighbourhood prevalence and *Eimeria* infections, even in the absence of a true relationship in the data.

### (d) Accounting for treated animals

As mentioned above, a subset of animals in the population received the anti-parasite drugs Ivermectin (which targets nematodes) or Vecoxan (which targets coccidial parasites like *Eimeria*). We have previously shown that Ivermectin is effective in reducing the intensity of nematode infection in wild wood mice, but the effect is transient, with treated individuals being reinfected within days of treatment [4,17]. For our analyses, we excluded animals that had been treated with Ivermectin as focal individuals, as it may confound our results. However, they were allowed to be considered as neighbours (i.e. they do occur on the grid, and so could potentially contribute to infection of focal individuals). We have not detected any observable effect of Vecoxan on either *Eimeria* species in these wood mouse populations (see electronic supplementary material, S2 for details) and so, given this lack of observable effect of treatment, we included Vecoxan-treated animals as both focal and neighbouring hosts in our analyses.

All analyses were undertaken in R (version 3.5.0), and all code and data files are available at the accompanying GitHub site (https://github.com/shaunkeegan/coinfection_spatial_scaling_paper).

## 3. Results

**Figure 2.**
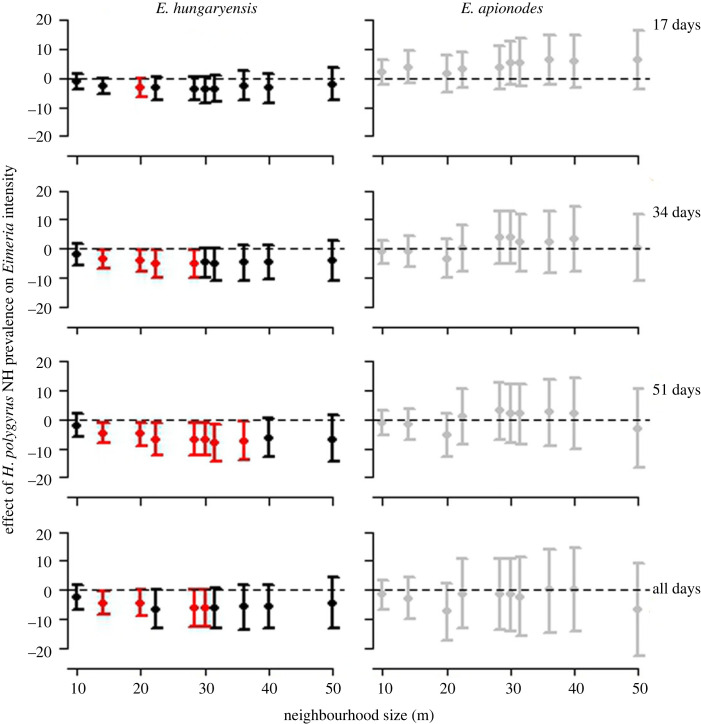
Neighbourhood analysis of parasite data from wood mice (*Apodemus sylvaticus*) showing the associations between the effect size from Bayesian GLMMs of the neighbourhood-level prevalence of the GI nematode *Heligmosomoides polygyrus* on focal individual infection intensity of (left column) *Eimeria hungaryensis* (known from previous experimental perturbations [4], to undergo strong within-host coinfection interactions with the *H. polygyrus*), and (right column) *E. apionodes* (known not to undergo strong within-host interactions with *H. polygyrus*) for increasing neighbourhood sizes (*x*-axes). Each row shows results for different time windows between captures of animals in the neighbourhood and subsequent capture of the focal individual. Figures show median model estimates (points) and 95% credible intervals (bars) for each neighbourhood size and time window; see Material and methods for full specification of model structure. Red points denote neighbourhood sizes for which the 95% credible intervals do not cross 0; black and grey points denote neighbourhood sizes for which 95% credible intervals do cross 0.

Our dataset comprised 986 captures of 252 wild wood mice with information on their *H. polygyrus* and *Eimeria* spp. infection statuses, and spatially explicit and time-referenced information on their capture locations. Our neighbourhood analysis revealed a general reduction in individual-level infection intensity of *E. hungaryensis* with increasing *H. polygyrus* infection prevalence among all other individuals in the surrounding neighbourhood (figure 2, left-hand panels). Hence, the greater the proportion of *H. polygyrus*-infected animals in a neighbourhood, the lower the average intensity of *E. hungaryensis* infections within each focal individual. Note that the statistical models controlled for the *H. polygyrus* infection status of the focals (as well as other potentially confounding factors: age, sex and time of year), and so these neighbourhood-level effects are over-and-above any direct effect of *H. polygyrus* on *E. hungaryensis* infections within the focals themselves.

Notably, the reduction in focal *E. hungaryensis* intensity with increasing neighbourhood *H. polygyrus* prevalence varied with both space (neighbourhood size) and time (length of the window between the capture of a neighbour and the focal's subsequent capture) (figure 2, left-hand panels). Spatially, the effects of neighbourhood *H.* polygyrus infection were most apparent approximately 15–40 m. At larger spatial scales (greater than 40 m), we found no association between neighbourhood *H. polygyrus* prevalence and *E. hungaryensis* intensity in focal hosts, consistent with our previous population-level analyses that found no associations between *H. polygyrus—E. hungaryensis* infections across the wider mouse population [13]. Temporally, neighbourhood-wide suppressive effects of *H. polygyrus* on focal *E. hungaryensis* intensity were strongest, and most widely observed, with a time window up to 51 days between neighbour and focal captures. Effect sizes were generally weaker, with credible intervals that overlapped zero, at both short time (up to 17 days) and long (all days) time windows.

**Figure 3.**
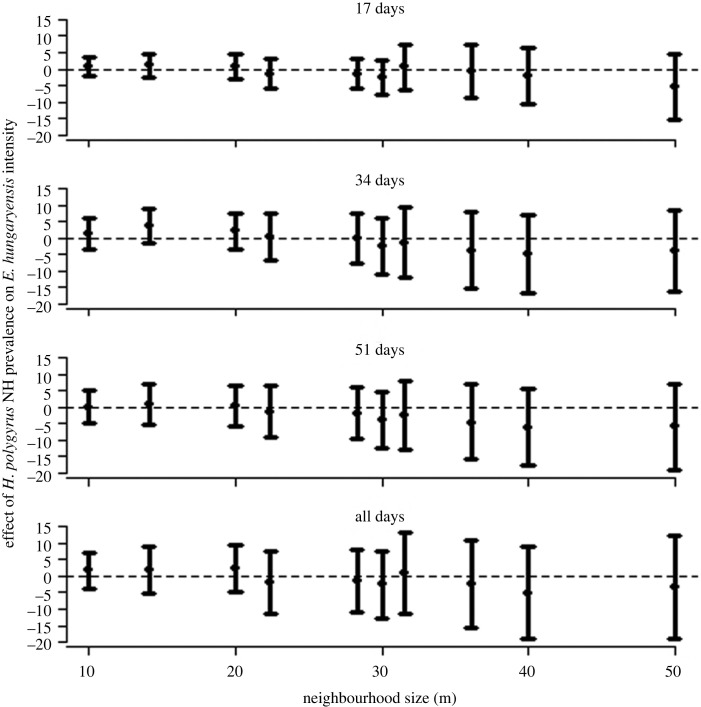
Results from neighbourhood analysis showing the associations between the effect size (from GLMMs) of the neighbourhood-level prevalence of the GI nematode *H. polygyrus* on focal individual *E. hungaryensis* infection intensity for increasing neighbourhood sizes, applied to a dataset that randomly assigned observed pairs of *H. polygyrus* and *E. hungaryensis* infection data from each animal to the spatial and temporal capture records of other animals in the dataset. Each panel shows results for different time windows between captures of animals in the neighbourhood and subsequent capture of the focal individual. Figures show median model estimates (points) and 95% credible intervals (bars) from Bayesian GLMMs of the effect of neighbourhood prevalence of *H. polygyrus* on individual-level intensity of *E. hungaryensis*, at each neighbourhood size, for each time window.

The above analyses suggest that the known, negative within-host effect of *H. polygyrus* on *E. hungaryensis* oocyst shedding leaves a signal of that interaction on the transmission dynamics of *E. hungaryensis* within the local neighbourhood. Comparison with the closely related species *E. apionodes*, which we know from previous drug-treatment experiments does not interact strongly with *H. polygyrus*, helps add veracity to these findings. As hypothesized, we see no signal of an effect of *H. polygyrus* neighbourhood prevalence on *E. apionodes* intensity at any neighbourhood size, or any time window duration (figure 2, right-hand panels). Furthermore, analysis of a permuted dataset in which real mouse capture locations and timings were randomly assigned *H. polygyrus* and *E. hungaryensis* infection data, found no signal of a relationship between *H. polygyrus* neighbourhood prevalence and *E. hungaryensis* infection intensities at any spatial or temporal scales (figure 3). Together, these neutral findings for the ‘natural control’ species *E. apionodes* and from the randomized dataset, help reassure that the findings of neighbourhood-wide suppression of *E. hungaryensis* infections were not due to a statistical artefact of the neighbourhood analysis method.

Finally, we explored alternative combinations of neighbourhood predictor variables (*H. polygyrus* mean burden) and response variables (*Eimeria* presence/absence). Generally, the effect of *H. polygyrus* neighbourhood mean abundance on *Eimeria* intensity (electronic supplementary material, figure S1) was weaker than that seen for neighbourhood *H. polygyrus* prevalence (figure 2), although there was a suggestion towards more suppressive effects (more negative effect sizes) for *E. hungaryensis* compared to *E. apionodes* (compare electronic supplementary material, figure S1, left- and right-hand panels). Credible intervals of effect sizes were noticeably larger when considering *H. polygyrus* mean burden as the predictor variable (electronic supplementary material, figure S1) than with *H. polygyrus* prevalence (figure 2), especially for the larger neighbourhood sizes and time windows. As hypothesized, based on previous experimental evidence [4], there was very little evidence of an effect of *H. polygyrus* prevalence (electronic supplementary material, figure S2) or mean burden (electronic supplementary material, figure S3) on *Eimeria* presence/absence, and even a general tendency of a positive association between neighbourhood *H. polygyrus* infection status and focal *Eimeria* presence/absence, for both *Eimeria* species, at larger neighbourhood sizes. This effect could be driven by a commonality in exposure of the nematode and protozoa through their shared transmission route (faecal-oral transmission via infective stages in the environment), a hypothesis supported by seeing the same tendency in both *Eimeria* species.

## 4. Discussion

Our study presents a spatially explicit analysis to assess the extent to which within-host interactions between coinfecting parasites alter between-host transmission dynamics across a range of spatial scales. We show that the previously recognized negative interaction between the GI nematode *H. polygyrus* and the protozoan *E. hungaryensis* [4,15] ripples out beyond individual hosts to reduce neighbourhood-wide transmission of *E. hungaryensis*. Specifically, the greater the proportion of hosts infected with *H. polygyrus* in the neighbourhood, the lower the intensity of *E. hungaryensis* infection in neighbouring hosts. However, the extent of this effect was limited both spatially and temporally around focal host individuals, likely representing the spatio-temporal scales of transmission. These findings suggest that the suppression of *E. hungaryensis* oocyst shedding by coinfecting *H. polygyrus* within individual hosts alters between-host transmission of the protozoan, but only over spatial scales that represent likely overlaps in space use of each mouse that result in transmission [18]. Confidence in our interpretation of these results is provided by the natural control of the closely related species *E. apionodes*, which has the same transmission mode and life cycle as *E. hungaryensis* but, due to its infection location in the mouse's gut (found primarily lower in the small intestine), does not interact strongly with *H. polygyrus* [4]. As hypothesized, our analysis found no signal of a suppressive effect of *E. apionodes* transmission by *H. polygyrus* infection over any neighbourhood size. This, together with our randomization analysis, helps reassure that the suppressive effects seen for *E. hungaryensis* are unlikely to be artefacts of our analytical technique, and so adds further weight to the validity of the suppressive effects seen between *H. polygyrus* and *E. hungaryensis*. To our knowledge, this is the first empirical demonstration of the occurrence and spatial extent of knock-on consequences of within-host coinfection interactions for parasite transmission in a natural host-parasite community.

The effects we detected were highly spatio-temporally dependent, and only occurred over restricted time scales and distances within the local neighbourhood of each host. Spatially, the effects of neighbourhood *H.* polygyrus infection were most apparent at over neighbourhoods from approximately 14–40 m. Wood mice in areas similar to ours have previously been estimated to have mean home range sizes of approximately 20 m radius [18]. Hence, the strongest effect sizes we found were over neighbourhood radii approximately 1–2 times their home range radius (i.e. spanning the range of potential overlaps of two adjoining home ranges). As such, the effects we see represent the likely spatial scale of transmission via overlaps in space use between neighbouring mice. Temporally, we saw strongest and more extensive neighbourhood effects with time windows between neighbours and focal animals of around 51 days; very short (17 days) or very long time windows resulted in few strong neighbourhood effects. This variation across time windows may be due to *Eimeria* developmental biology. *Eimeria* oocysts undergo a period of development in the environment before becoming infectious, typically thought to be approximately one week based on related species [19], and may remain infective for several months [20]. Hence very short time windows may be too small to allow sufficient accumulation of infective stages to detect neighbourhood effects. However, long time windows will include a wide range of temporal overlaps in neighbour and focal space use, potentially obscuring the signal of neighbourhood effects on focal infections. Hence, 51 days may represent an appropriate window to balance these effects, revealing the strongest and most spatially extensive neighbourhood effects in our analyses. We also note that evidence of transmission modification was strongest and most consistent when looking at the effect of neighbourhood-wide *H. polygyrus* prevalence on focal individual *E. hungaryensis* intensity; other measures of neighbourhood predictor and focal response variables were less clear. In particular, no effect was seen with *E. hungaryensis* presence/absence as the response variable; this matched our prediction, given the previous lack of support for an individual-level effect of deworming on *E. hungaryensis* presence/absence [4]. There was some evidence of negative associations between neighbourhood-wide *H. polygyrus* abundance on *E. hungaryensis* intensity, but effects were much weaker and more variable than seen for *H. polygyrus* prevalence. This is perhaps to be expected as mean abundance is generally considered an incomplete or inaccurate measure of macroparasite occurrence across a host (sub-)population, particularly when assessed indirectly via faecal egg counts and/or sample sizes are limited, as here [21–24].

Our findings have significant implications for the use of population-level analyses to screen for potential coinfection interactions between parasites. Neighbourhood sizes beyond 30–40 m, and time scales that incorporated all previous captures, resulted in the magnitude of the suppressive effect on *E. hungaryensis* intensity being diminished. This lack of association between *H. polygyrus* and *E. hungaryensis* at large spatial and temporal scales corresponds with previous analyses that have failed to detect a signal of the *H. polygyrus–E. hungaryensis* interaction at the level of the whole population (i.e. grid-level analyses) [13]. Combining data across all spatial and temporal scales will combine a wide range of shedding-to-infection processes, obscuring meaningful associations between neighbour and focal infections (as evidenced by the generally larger credible intervals seen at larger neighbourhood sizes and time windows; figure 2; electronic supplementary material, figures S1–S3). Hence, these findings suggest that snapshots of parasite associations at the full population level (i.e. combining data over relatively large spatial and temporal scales) will likely be unable to reliably infer within-host interactions between parasites. However, examining parasite associations at smaller spatial and temporal scales, which more appropriately reflect local host movement, space use, and parasite transmission biology, will more accurately reveal underlying interactions, while not generating spurious inferences of interactions between species that do not interact strongly (e.g. *H. polygyrus* and *E. apionodes* [4]). Our approach, which explicitly considers both spatial and temporal scales, therefore has potential to identify possible coinfection interactions that otherwise are only detectable by experimental intervention. More broadly, these points highlight the importance of adopting a joint spatio-temporal approach, rather than either purely spatial or purely temporal, in elucidating key aspects of parasite transmission dynamics in natural systems [18].

The spatial heterogeneities revealed here arise from two standard epidemiological features of any host-parasite system. Firstly, hosts are not homogeneously mixed, for example due to territoriality, resource distribution, mating behaviours etc. Secondly, parasites are not necessarily homogeneous in a host population, for example due to variations in individual host immune state and host behaviour or environmental heterogeneities which affect external parasite stages [18], creating both hot- and cold-spots of parasite occurrence even in well-mixed host populations. Coupling this natural variation in parasite distribution with a non-uniform host population suggests that spatial heterogeneities are likely to be an important part of most host-parasite communities, and hence a driver of coinfection-mediated transmission modification.

Overall, we have shown that within-host interactions between coinfecting parasites affect local transmission dynamics, leaving a signal of the within-host interaction at spatial scales that ripple out beyond the individual host through a process of transmission modification, impacting infection risk and intensity for the neighbouring hosts. However, these effects tend to be highly spatio-temporally dependent, probably reflecting the spatial and temporal scales of transmission and the movement and space-use patterns of the hosts. There are many human, livestock and wildlife disease systems where there are well-documented within-host interactions among coinfecting parasites, and a growing body of research has examined the consequences of those interactions for the success and impact (beneficial or detrimental) of disease treatment approaches on individual host health [25,26]. A major implication of our work is that there could be knock-on, between-host consequences of such treatments, particularly in communities experiencing high coverage mass drug administration, for localized transmission dynamics of non-target parasites, with implications for altered infection risk among non-treated individuals in the wider neighbourhood.

## Data Availability

Data and code are available on Dryad [27] and Zenodo [28]. Data and R code used for this paper are available from https://github.com/shaunkeegan/coinfection_spatial_scaling_paper. Additional data are provided in electronic supplementary material [29].
